# Intraoperative Fluorescence Imaging for Personalized Brain Tumor Resection: Current State and Future Directions

**DOI:** 10.3389/fsurg.2016.00055

**Published:** 2016-10-17

**Authors:** Evgenii Belykh, Nikolay L. Martirosyan, Kaan Yagmurlu, Eric J. Miller, Jennifer M. Eschbacher, Mohammadhassan Izadyyazdanabadi, Liudmila A. Bardonova, Vadim A. Byvaltsev, Peter Nakaji, Mark C. Preul

**Affiliations:** ^1^Department of Neurosurgery, St. Joseph’s Hospital and Medical Center, Barrow Neurological Institute, Phoenix, AZ, USA; ^2^School of Life Sciences, Arizona State University, Tempe, AZ, USA; ^3^Laboratory of Neurosurgery, Irkutsk Scientific Center of Surgery and Traumatology, Irkutsk, Russia; ^4^Irkutsk State Medical University, Irkutsk, Russia; ^5^University of Arizona College of Medicine – Phoenix, Phoenix, AZ, USA

**Keywords:** 5-ALA, confocal, endomicroscopy, fluorescein, fluorescence-guided surgery, fluorescent probe, glioma, ICG

## Abstract

**Introduction:**

Fluorescence-guided surgery is one of the rapidly emerging methods of surgical “theranostics.” In this review, we summarize current fluorescence techniques used in neurosurgical practice for brain tumor patients as well as future applications of recent laboratory and translational studies.

**Methods:**

Review of the literature.

**Results:**

A wide spectrum of fluorophores that have been tested for brain surgery is reviewed. Beginning with a fluorescein sodium application in 1948 by Moore, fluorescence-guided brain tumor surgery is either routinely applied in some centers or is under active study in clinical trials. Besides the trinity of commonly used drugs (fluorescein sodium, 5-aminolevulinic acid, and indocyanine green), less studied fluorescent stains, such as tetracyclines, cancer-selective alkylphosphocholine analogs, cresyl violet, acridine orange, and acriflavine, can be used for rapid tumor detection and pathological tissue examination. Other emerging agents, such as activity-based probes and targeted molecular probes that can provide biomolecular specificity for surgical visualization and treatment, are reviewed. Furthermore, we review available engineering and optical solutions for fluorescent surgical visualization. Instruments for fluorescent-guided surgery are divided into wide-field imaging systems and hand-held probes. Recent advancements in quantitative fluorescence-guided surgery are discussed.

**Conclusion:**

We are standing on the threshold of the era of marker-assisted tumor management. Innovations in the fields of surgical optics, computer image analysis, and molecular bioengineering are advancing fluorescence-guided tumor resection paradigms, leading to cell-level approaches to visualization and resection of brain tumors.

## Introduction

Malignant glioma is a highly invasive, heterogeneous, complex, and fatal tumor type, the extent of which is not precisely identifiable by modern imaging techniques. Despite all of the current treatment modalities for malignant gliomas, such as microsurgery, chemotherapy, and radiotherapy, there is no definitive treatment. Nonetheless, the maximum extent of surgical resection is associated with a longer recurrence-free period and overall survival of patients with glioblastomas (1, 2), low-grade gliomas (3), meningiomas (4), and other intracranial malignancies. Therefore, the initial treatment goal should be the maximal removal of the tumor mass. Tumor mass resection is guided intraoperatively by anatomically registered images (usually CT and MRI) incorporated into a stereotactically based image-guided surgery platform. Such a surgical strategy becomes a balance of aggressive tumor removal while producing no new or further permanent neurological deficit. Although there are characteristics of images from CT and MRI that indicate what tumor, necrosis, or edematous cortex is, the main focus of surgery is achieving maximal resection of the invading tumor front. In light of this, researchers have endeavored to make any invisible part of the tumor visible using advanced imaging techniques.

Advances in imaging began with the philosophies of cerebral localization and function, while techniques for improving precision and the customization of brain tumor surgery can be traced to the late nineteenth century. The evolution of imaging techniques in neurosurgery began with the first attempts at craniometric localization of intracranial lesions (5). The introduction of X-rays in neurosurgery in 1896 by Krause and Cushing (6), pneumoencephalography in 1919 by Dandy (7, 8), and cerebral angiography specifically for brain tumors by Moniz in 1927 (9, 10) were the first steps in preoperative imaging diagnosis of brain tumors, which was previously possible only by clinical neurological examination. Intraoperative stimulation in awake patients to increase the safety of tumor resection was performed by Thomas and Cushing (11). This stimulation was possible due to Cushing’s previous experience in mapping the motor cortex of primates in 1902 in the physiology laboratory of Sherrington (12). However, the origins of intraoperative neurophysiology for functional localization have roots in the works of Betz (13), Ferrier (14), Fritsch and Hitzig (15), and Clark and Horsley (16). Since the beginning of the twentieth century, several neurosurgeons, most notably Penfield in 1928, have used intraoperative brain stimulation extensively to map the cortex to guide brain tumor resection and surgical treatment of epilepsy (17). Building on earlier work, intraoperative electrophysiological monitoring and cortical and subcortical mapping performed with the patient conscious remain state-of-the-art methods to elicit functions of brain areas and define and personalize safe boundaries of tumor resection (18, 19).

Techniques for visually identifying the tumor mass began in the mid-twentieth century. The application of the fluorescent dye fluorescein sodium to highlight tumor tissue during its removal was introduced in neurosurgery by Moore et al. in 1948 (20), decades before computed tomographic (CT) scanning was introduced into broad clinical practice (1973) (21–23). Fluorescein sodium was in use even earlier than the first operative microscopes used by neurosurgeons and was pioneered by Kurze in 1957 (24). However, fluorescent dye technology did not gain widespread acceptance due to the high rate of background fluorescence from normal brain tissue and the shortcomings of visualization technologies (25). Fluorescein injection for cerebrovascular and tumor surgery was studied in detail by Feindel in the 1960s (26). A major step after the introduction of CT with contrast injection (23) was gadolinium-enhanced MRI, introduced around 1987, that allowed even more precise tumor mass visualization and precise anatomical co-registration for planning surgery (27). Infrared frameless neuronavigation systems resulting from developments in stereotactic and computer technologies were rapidly adopted in neurosurgical operating rooms in the late 1980s (28–30). The virtual linkage of neuroimaging and intraoperative anatomy allowed a precision of nearly 2 mm, selection of the best approach trajectory (31), and radically improved the surgeon’s intraoperative orientation. The main drawback of neuronavigation remains brain shift [1 cm on average (32)] as a consequence of opening the cranium, which significantly limits the accuracy of determining an infiltrative tumor border (33). Despite software advances (32), intraoperative ultrasound (34, 35), and intraoperative MRI corrections (36), the current (2015) technologies do not provide the desired accuracy for consistent, precise, and extensive resection (37). The main drawback continues to be that MR and CT image characteristics are not directly indicative of regional tissue type and cannot provide clinically applicable imaging at or near cellular resolution.

Accurate visualization of brain tumors marked by fluorescent probes and even residual tumor cells is possible with emerging new technologies. These emerging technologies are expected to become state-of-the-art tools to maximize customized brain tumor treatment. These technologies are the logical extension of the evolution of the search for precision in brain tumor surgery. Such technologies will allow real-time imaging interrogation of the brain during surgery at the cellular resolution to maximize or tailor brain tumor resection.

This review summarizes recent achievements and future perspectives of clinical, laboratory, and translational studies that bring fluorescence-guided neurosurgery to the cellular level, thereby allowing for individualized brain tumor resections, representing a crucial breakthrough in this field.

## Fluorescent Dyes in Neurosurgery

In the last decade (2006–2016), the number of fluorescent stains and cellular tags used in preclinical studies has increased significantly, with many novel fluorophores awaiting approval for clinical use. The fluorescent probes and dyes discussed in this review are summarized in Table 1 (25, 38–74). Three fluorescent contrast agents that have been studied and used in human neurosurgical procedures are fluorescein sodium, indocyanine green (ICG), and 5-aminolevulinic acid (5-ALA), although not all are approved by regulatory committees in all countries. Other fluorophores (including acridine orange, acriflavine, cresyl violet, and sulforhodamine 101) have been used for pulmonary, gastrointestinal, or gynecologic procedures and in *ex vivo* brain biopsies. They have not been used directly in the human brain. Fluorescent probes and labels are classified based on the actual fluorescent molecule (i.e., intrinsic and extrinsic endogenous fluorophores) and excitation/emission profile and can be further categorized by their mechanism of action:

1. Passive fluorescent probes (ICG, fluorescein sodium, and other stains);
2. Metabolic probes (5-ALA, activatable probes); and
3. Targeted probes.

**Table 1:** **Summary of published preclinical and early clinical data on probes and imaging equipment for potential personalized fluorescence-guided brain tumor surgery**.

Name of probe	Reported excitation wavelength	Reported reading emission wavelength	Used equipment	Species tested	Advantages	Disadvantages	Mode of administration and time to imaging (unless noted otherwise)
**Targeted probes**							
IRDye 800CW-labeled VEGF (38) (Bevacizumab)	675 and 745 nm (*in vivo*)	800 nm (*in vivo*)	IVIS Spectrum (PerkinElmer, Inc.) Multispectral Fluorescence Camera System (Institute for Biological and Medical Imaging, Technical University, Munich, Germany and SurgOptix Inc., Redwood Shores, CA, USA), *in vivo* Olympus Fluoview 300 Confocal Scan Box mounted on an Olympus IX 71 inverted microscope (Olympus America Inc.), *ex vivo* Pearl Imaging System (LI-COR Biosciences) *in vivo*	Xenograft mice model (human ovarian, breast, and gastric cancers)	Distinguish submillimeter lesions intraoperatively. Longer lasting and more accurate signal for VEGF and EGFR2 than ICG alone. Bevacizumab-800CW fluorescence detection in extracellular matrix, trastuzumab-800CW fluorescence detection on tumor cell surface	Long half-life for detecting tumors. Long elimination time	IV, 6 days (optimal time)

IRDye 800CW-labeled human EGFR 2 [Trastuzumab (38); Erbitux (39)]	675 and 745 nm (*in vivo*); 685 and 785 nm	800 nm (*in vivo*); 720 and 820 nm	IVIS Spectrum (PerkinElmer, Inc.) Multispectral Fluorescence Camera System (Institute for Biological and Medical Imaging, Technical University and SurgOptix Inc.), *in vivo* Olympus Fluoview 300 Confocal Scan Box mounted on an Olympus IX 71 inverted microscope (Olympus America Inc.), *ex vivo* Pearl Imaging System (LI-COR Biosciences) *in vivo*	Xenograft mice model (human ovarian, breast, and gastric cancers); Xenograft mice model (human breast cancer lymph metastasis)	Distinguish submillimeter lesions intraoperatively. Longer lasting and more accurate signal for VEGF and EGFR2 than ICG alone. Bevacizumab-800CW fluorescence detection in extracellular matrix, trastuzumab-800CW fluorescence detection on tumor cell surface	Long half-life for detecting tumors. Long elimination time	IV, 3–6 days (optimal time); 3 h for lymph node visualization

IRDye 800CW-labeled anti-EGFR nanobody 7D12 (40, 41)	760 nm; 656–678 nm; 745–779 nm	774 nm; 700 nm; 800 nm	IVIS Lumina System (PerkinElmer, Inc.) with ICG filter sets FLARE imaging system (Beth Israel Deaconess Medical Center) IVIS Spectrum (PerkinElmer, Inc.)	Xenograft mice (human epidermoid carcinoma); xenograft mice (human metastatic oral squamous cell carcinoma)	Better tumor penetration and distribution of nanobody probe *in vivo* (vs. cetuximab full antibody). Significantly higher tumor to background fluorescence (vs. cetuximab full antibody)	Not mentioned	IV, 30 min (earliest); 2 h (optimal); or 24 h (optimal)

IRDye 680RD labeled EGFR inhibitor (cetuximab) (42)	620 nm	650–800 nm	Odyssey Infrared Imaging System (LI-COR Biosciences, Lincoln, Nebraska), *ex vivo*	Xenograft mice (human U251 glioma)	Higher affinity for tumor than anti-EGFR targeted affibody used in same study	Concentration of antibody in tumor focused primarily in the center	IV, 1 h

IRDye 800CW-labeled anti-EGFR targeted affibody (42)	720 nm	730–900 nm	Odyssey Infrared Imaging System (LI-COR Biosciences), *ex vivo*	Xenograft mice (human U251 glioma)	Smaller size molecule results in better penetration of BBB. Higher concentration in outer tumor than antibody	30 times lower affinity than antibody and a shorter plasma half-life	IV, 1 h

IRDye 800CW-labeled chemokine stromal cell derived factor-1 (SDF-1) (43)	685 and 785 nm	702 or 789 nm	Pearl Imaging System (LI-COR Biosciences), *in vivo*	Xenograft mice (A764 human glioma, MCF-7 human breast cancer)	Detected as low as 500 cells *in vitro*. Specific for tumor cells. Signal persisted for days	Labeled bone marrow, transient non-specific labeling during first 24 h was observed in the liver and skull	IV, 1-h visualization of tumors and background structures; 24–92 h background fluorescence diminished, tumors remained clearly visible

IRDye 800CW-labeled anti-CD105 monoclonal antibody (angiogenesis related) (44)	778 nm	806 nm	Pearl Imaging System (LI-COR Biosciences) *in vivo* and *in vitro*	Mice with 4T1 mouse breast cancer; human MCF-7 breast cancer cells in cultures	Tumor could be visualized as early as 30 min post-injection; may be used in the clinic for imaging tumor angiogenesis	CD105 expression is observed only on actively proliferating tumor endothelial cells	IV, 30 min (early); 16 h (optimal)

Cy5.5-labeled EGFR inhibitor (cetuximab) (45)	683 nm (max); 630–670 nm (range used in experiment)	707 nm (max); 685–735 nm (range used in experiment)	Leica MZFL3 stereo research microscope (Leica Microsystems, Bannockburn, IL, USA) fitted with a GFP and Cy5.5 filter and an ORCA ER charge-coupled device camera (Hamamatsu, Bridgewater, NJ, USA) eXplore Optix time-domain fluorescence imaging system (ART/GE Healthcare, Princeton, NJ, USA)	Cell cultures: UM-SCC-1, FaDu, CAL 27, and AB12; xenograft mice model (human head and neck squamous cell carcinoma cell lines SCC-1, FaDu, CAL 27); mice with mouse mesothelioma	Can be used to detect tumors *in vivo*	EGFR expression did not correlate with the fluorescent intensity	IV, 48–72 h (optimal)

Alexa-680 labeled insulin-like growth factor 1 receptor (IGF1 R) (AVE-1642-conjugated Alexa 680) (46)	575–605 nm	645–850 nm	Maestro Imaging System (CRI), *in vivo* Olympus Fluoview FV500 laser scanning confocal system (Olympus America Inc.)	Xenograft mice model (MCF-7 human breast cancer cells)	Can detect the downregulation of IGF1R after treatment with a monoclonal antibody	Further studies required to determine the amount of background fluorescence produced by IGF1R	1 day (earliest); 2 days (clear imaging)
Folate–fluorescein isothiocyanate probe (for folate receptor) (47)	495 nm	520 nm	Intraoperative Multispectral Fluorescence Camera System (Institute for Biological and Medical Imaging, Technical University)	Humans with ovarian cancer	High specificity for labeling FR-alpha expressing cells. Real-time image-guided excision of fluorescent tumor deposits of size <1 mm was feasible	Four patients experienced mild discomfort in the upper abdominal region after injection	Imaging completed 2–8 h after injection

BODIPY FL-labeled PARP inhibitor (Olaparib) (48)	503 nm	515 nm	Maestro Imaging System (CRI)	Xenograft mice model (U87 MG and U251 MG human glioblastomas)	High specificity for the DNA repair enzyme PARP1 with therapeutic effect. Promising new targeted antitumor drug, which is already in clinical trials. High tumor-background fluorescent ratio. Toxicity profile is known and similar to Olaparib	Not mentioned	60–180 min (optimal)

Liposomes with RGD peptide and the neuropeptide SP, gadolinium, Indium-111, Rhodamine-B (49)	554 nm	576 nm	Zeiss LSM 510 Microscope (Carl Zeiss Meditec AG, Jena, Germany)	Cultured mouse fibroblast cells with U87 MG human glioblastoma and M21 human melanoma tumor cells (*in vitro*)	Combination of radioactive, fluorescent, and magnetic resonance imaging signaling; multifunctionality of liposomes as a carrier of different probes	Moderate tumor uptake	*In vitro* fluorescence microscopy was completed after tumor cells were grown in mouse fibroblast culture

ZW800-1 zwitterionic NIR fluorophore (50, 51)	750 ± 25 nm; 773 nm	810 ± 20 nm; 790 nm	FLARE Imaging System (Beth Israel Deaconess Medical Center) FLARE Imaging System (Beth Israel Deaconess Medical Center) Pearl Small Animal Imaging System (LI-COR Biosciences)	Xenograft mice model (M21 human melanoma, Lewis lung carcinoma, HT-29 human colorectal adenocarcinoma)	Higher tumor-to-background ratio than IRDye800-CW and Cy5.5	Wash-out of dye from tumors started occurring at 4 h (dye still present at 24 h)	4 h, low visibility at 4 h, highest visibility from 24 to 72 h

M13-stabilized single-walled carbon nanotubes (SBP-M13-SWNTs) (52)	808 nm	950–1400 nm	Liquid nitrogen-cooled OMA V 2D InGaAs array detector with a 256 × 320 pixel array (Princeton Instruments) coupled with SWIR-25 NIR camera lens (Navitar, Rochester, NY, USA)	Xenograft mice model (OVCAR8 human ovarian epithelial carcinoma)	Stable and showed 10 times more selective fluorescent staining of ovarian tumor cells than same construct without targeting peptide. Nanotube fluorescence intensity relative to background (5.5 ± 1.2) was superior to same construct labeled with other NIR AlexaFluor750 dye (3.1 ± 0.42) or FITC (0.96 ± 0.10)	Study did not assess possible penetration of the probe into the brain	24 h

Fluorescent gold nanoparticles conjugated with diatrizoic acid and AS1411 aptamer (53)	400 nm	620 nm (max)	Ultra-VIEW RS Confocal System (PerkinElmer, Inc., Waltham, MA, USA) IVIS (PerkinElmer, Inc.)	Xenograft mice model (human lung adenocarcinoma) separate MCF-7 cell assay	Specific binding to tumor cells due to AS1411 aptamer, which targets nucleolin. Allowed X-ray visualization due to high electron density of gold nanoparticles	Small sample size (*n* = 6)	30 min

Lymphoma-specific fluorescent (Alex488) switchable TD05 aptamer (54)	489 nm	505–535 nm	Zeiss 710 laser Scanning Confocal Microscope (Carl Zeiss Meditec AG) equipped with a 40×/1.2NA water emersion objective (*ex vivo*)	Xenograft rat model (U251 human glioma and Ramos human CNS lymphoma)	Probe could rapidly and specifically identify human B cell lymphoma in biopsies. System would be useful for discriminating non-operative CNS B-cell lymphoma from malignant glioma rapidly after biopsy	*Ex vivo* only study	Total antibody staining time was 24 h and aptamer staining time was 1 h (*ex vivo*)

Chlorotoxin (CTX) conjugated to ICG (BLZ-100) (55)	785 nm	Near-infrared spectrum	Custom imaging system: 16-mm VIS-NIR Compact Fixed Focal Length Lens (Edmund Optics, Barrington, NJ, USA) coupled 785-nm StopLine single-notch filter, NF03–785E-25 (Semrock, Rochester, NY, USA)	Xenograft mice model (LN229 human glioblastoma)	High affinity to human gliomas	Not mentioned	48 h

5-Carboxyfluorescein (FAM)-labeled fluorescent probe consisting of tLyP-1 small peptide targeted to the neuropilin receptors (FAM-tLyP-1) (56)	Blue light	Not given	Kodak *In Vivo* Imaging System F (Kodak, Rochester, NY, USA)	Xenograft mice model (U87MG human glioblastoma)	Selective uptake. May have advantages over CTX-Cy5.5 probe due smaller size	Fluorescein labeling was less than ideal, could be exchanged for more intense fluorophore	1 h

**Activity-based probes**							
Modified hydroxymethyl rhodamine green (gGlu-HMRG) (57)	488 nm	505–530 nm	In-house-made portable fluorescence camera for *ex vivo* tumor specimens Zeiss LSM510 Microscope (Carl Zeiss Meditec AG)	Human breast cancer tissue samples; breast cancer cell culture	High sensitivity and spatial resolution Fast processing: evaluation can be done within 5 min after probe application	In breast cancer, this method cannot distinguish malignant and benign regions	5 min

MMPSense 750 FAST (MMP-750) (58)	749 nm	775 nm	Surgical Navigation System (Institute of Automation, Chinese Academy of Sciences, Beijing, China) (59)	Mice with 4T1-luc breast cancer tumors	Imaging method offered precise detection of the orthotopic breast tumors and metastases intraoperatively in real time	Not mentioned	IV, 6 h (fluorescent signal observed); 24–36 h (optimal fluorescent signal)

Caspase-sensitive nano-aggregation fluorescent probe (C-SNAF) (60)	635 ± 25 nm	670–900 nm	Maestro Hyperspectral Fluorescent Imaging System (CRI)	Xenograft mice model (subcutaneous HeLa tumors)	Highly feasible for imaging of drug-induced tumor apoptosis *in vivo*, signal strengthens as tumor cells die	Not mentioned	IV, 1 h

**Nanoparticles**							
Polyacrylamide-based nanoparticles loaded with ICG or Coomassie blue dye (61)	647 nm	675–725 nm	Olympus IX70 confocal microscope (Olympus America, Inc.) Ultra-VIEW Confocal Laser Scanning Microscope (PerkinElmer, Inc.)	Cell cultures: 9L rat gliosarcoma, MDA-MB-435 human melanoma, MCF-7 human breast cancer	Produced visible color change in tumor cell lines	Significant non-specific binding was observed	Imaged after 2 h of incubation

Iron oxide magnetic NH_2_-CLIO nanoparticles labeled with Cy5.5 (Cy5.5-CLIO) (62)	Not given	Not given	Custom-built surface reflectance imaging system (Siemens Medical Systems, Erlangen, Germany), *in vivo* Zeiss LSM 5 Pascal (Carl Zeiss Meditec AG, Jena, Germany), *ex vivo*	Rat 9L gliosarcoma tumor model	Clear tumor border demarcation Co-localization based on MRI imaging Elimination more predictable than other nanoprobes	Not as accurate as target probes for *in vivo* tumor cell visualization	IV, 24 h

Cyto647 labeled anti-EGFR antibody-conjugated SERS-tagged gold nanoparticles (antibody-Panitumumab) (63)	642 nm (Olympus); 785 nm (Raman)	700–775 nm	Olympus IX81 inverted fluorescence microscope (Olympus America, Inc.) Hamamatsu Back-Thinned EM-CCD camera, 9100-13 (Hamamatsu, Bridgewater, NJ, USA) Spinning Disk Confocal Scanning Raman Microscope (Renishaw, Wotton-under-Edge, UK)	*In vitro* cell cultures: rat gliosarcoma cell line 9L; rat C6 glioma; human GBM cells U87, A172, U251, U373; normal fetal human astrocytes; primary oligodendroglioma tumor cells BT2012036; and GBM adherent stem cell line GLINS1	Selective uptake by tumor cells; unlike other fluorescent dyes, SERS nanoparticles have enhanced photostability	Not mentioned	Not applicable

**Others**							
5-ALA that metabolically converts into fluorescent PpIX	400–410 nm violet	620–720 nm red	VWCE Zeiss Pentero Microscope (Carl Zeiss Surgical GmbH)	Studies in human (25) and in animals (64)	Studies have shown increased extent of tumor resection with PpXI guided surgery; useful for brain tumor biopsy	Disruption of BBB necessary for fluorophore accumulation (can decrease/vary contrast) Low fluorescence intensity in low-grade gliomas	Oral, IV, 2 h (65)

Indocyanine green (ICG)	780 nm	>795 nm	VWCE Zeiss Pentero Microscope (Carl Zeiss Surgical, GmbH) Zeiss LSM710 (Carl Zeiss Surgical, GmbH)	Mice with GL261 mouse glioma (66)	Extensively studied; hand-held confocal endomicroscope and LSM showed ICG selectively stained glioma cells in mouse model (66)	ICG visualization can only be displayed on a monitor	IV, 15 min
				Human (67)	Intraoperative administration at end of 5-ALA guided resection may show additional tumor tissue (67)		

Fluorescein sodium (68)	494 nm	521 nm	VWCE Zeiss Pentero Microscope (Carl Zeiss Surgical GmbH) LSM710 (Carl Zeiss Surgical, GmbH)	Human	Convenience for surgeon, surrounding tissue has more natural color	Rapid photobleaching, non-specific accumulation of fluorescein along the margins of resection. Possible extravasation along with edema	IV, 5 min (65)

CLR1501 (69)	500 nm	517 nm	Nikon A1RSi Confocal Microscope (Nikon, Minato, Tokyo, Japan); IVIS Spectrum system (PerkinElmer, Inc.)	Xenograft mouse model (U251 human glioblastoma, 22T, 22CSC, 33CSC, 105CSC patient derived glioblastoma)	Tumor-to-brain fluorescence ratio similar to 5-ALA	Tumor must be visualized on separate monitor	IV, >4 days

CLR1502 (69)	760 nm	778 nm	IVIS Spectrum system (PerkinElmer, Inc.) Fluobeam 800 (Fluoptics, Grenoble, France) Leica OH4 intraoperative microscope with FL800 attachment (Leica Microsystems, Bannockburn, IL, USA)	Xenograft mouse model (U251 human glioblastoma, 22T, 22CSC, 33CSC, 105CSC patient derived glioblastoma)	Tumor-to-brain fluorescence ratio superior to 5-ALA	Tumor must be visualized on separate monitor	IV, >4 days

CH1055 (70)	~750 nm	1055 nm	In-house-built NIR spectroscopy instrument with Acton SP2300i spectrometer (Princeton Instruments, Trenton, NJ, USA) and Princeton OMA-V liquid-nitrogen-cooled InGaAs linear array detector (Princeton Instruments)	Xenograft mice model (U87MG human glioblastoma)	High tumor-to-background signal ratio Possibility for precise image-guided tumor removal in the model 90% excreted through the kidneys within 24 h	Tumor must be visualized on separate monitor	IV, 6 h (tumor is clearly visible); 72 h (optimal)

Acridine orange (64)	488 nm	505–700 nm (LSM); 505–585 (VWCE)	VWCE Zeiss Pentero Microscope (Carl Zeiss Surgical GmbH) LSM710 (Carl Zeiss GmbH)	Mice with GL261 glioma; swine normal brain	Suitable for rapid intraoperative *ex vivo* analysis of glioma tissue	Cannot be used in the brain due to toxicity profile	Topical application, immediately

Acriflavine (64)	405 nm (LSM); 488 (VWCE)	505–585 nm	VWCE Zeiss Pentero Microscope (Carl Zeiss Surgical GmbH) LSM710 (Carl Zeiss GmbH)	Mice with GL261 glioma	Suitable for rapid intraoperative *ex vivo* analysis of glioma tissue	Cannot be used in the brain due to toxicity profile	Topical application, immediately

Cresyl violet (64)	561 nm (LSM); 488 nm (VWCE)	620–655 nm (LSM); 505–585 nm (VWCE)	VWCE Zeiss Pentero Microscope (Carl Zeiss Surgical GmbH) LSM710 (Carl Zeiss GmbH)	Mice with GL261 glioma	Highlights tumor boundaries *ex vivo*	No current *in vivo* brain application Low signal-to-noise ratio	Topical application, 10 min

Sulforhodamine 101SR101 (64)	561 nm (LSM); 488 nm (VWCE)	585–615 nm (LSM); 505–750 nm (VWCE)	VWCE Zeiss Pentero Microscope (Carl Zeiss Surgical GmbH) LSM710 (Carl Zeiss GmbH)	Xenograft rat model (U251 human glioma)	Strongly labeled cells within the tumor and astrocytes within normal brain	Non-specific	1 h

Demeclocycline (72)	402 nm	~520 nm	Custom confocal laser scanning microscope	Human low- and high-grade glioma tissues	Highlights tumor cells *ex vivo* and correlates with histology. Limited data suggest specificity for tumor cells (73)	Non-specific	Topical application, timing not reported

Methylene blue (74)	642 nm	~690 nm	Custom confocal laser scanning microscope	Human meningioma, glioma, and adenocarcinoma tissues	Highlights tumor cells *ex vivo*	Non-specific	Topical application, timing not reported

*BBB, blood–brain barrier; VEGF, vascular endothelial growth factor; EGFR, epidermal growth factor receptor; SERS, surface-enhanced Raman scattering, a nanoparticle tagging method to increase signal detection; FMI, fluorescence molecular imaging; BCS, breast cancer surgery; LSM, laser scanning microscope; VWCE, visible wavelength confocal endomicroscope (Optiscan 5.1) (71)*.

**Figure 1:**
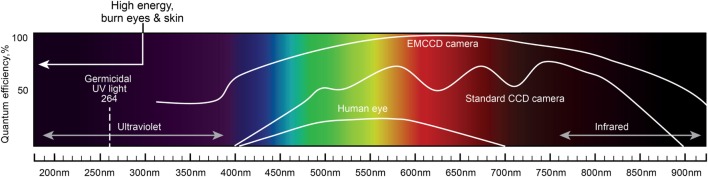
**A schematic diagram of the light spectrum and corresponding wavelengths**. Quantum efficiency of the human eye, standard CCD camera, and EMCCD camera are plotted together to show the differences in the covered wavelengths and the sensitivity to light. Light with shorter wavelengths has higher energy than light with longer wavelengths. Light wavelengths below 300 nm may burn eyes and skin. UV light of 264 nm is germicidal. Longer wavelengths (infrared) have greater tissue penetration properties. EMCCD, electron multiplying charge-coupled device; CCD, charge-coupled device. Used with permission from Barrow Neurological Institute, Phoenix, AZ, USA.

One of the most important characteristics of the probes is their ability to accumulate in tumor tissues in high concentrations. In the case of brain tumors, the blood–brain barrier (BBB) influences the delivery of probes that are not lipophilic or have a molecular weight more than 400–600 kDa (75). Based on their physical properties, photons with longer wavelengths in the near-infrared (NIR) spectrum have greater tissue penetration and thus are advantageous for visualizing obscure residual tumor tissue or cells (Figure 1). However, fluorophore phototoxicity caused by the generation of reactive oxygen species (ROS) may be harmful to healthy cells. The principle of phototoxicity is also used in combination with fluorescence-guided tumor resection and photodynamic therapy (PDT). Most fluorescence is associated with the production of some ROS and PDT effects (76). The combination of fluorescence-guided resection and post-resection cavitary PDT with strong photosensitizers may have a synergistic effect and has already shown promising results in several clinical trials (77, 78). Nonetheless, this approach and the exact methodology regarding the choice of a photosensitizer, excitation wavelengths, dosages, and other parameters remain to be defined.

In this section, we discuss fluorescent agents that are used or could potentially be used for fluorescence-guided resection and intraoperative diagnosis of brain tumors.

### Indocyanine Green

#### Characteristics

Indocyanine green is a small water-soluble molecule with molecular weight of 744.96 Da. ICG is excited at the wavelength of about 780 nm, and it emits fluorescence in the 700- to 850-nm range, which is not visible to the naked eye. After IV administration, ICG binds to plasma proteins and is cleared by the liver. In brain tumor surgery studies, 5- to 25-mg ICG concentrations were used (79, 80), and the observed duration of fluorescence was limited, with a peak at about 10 min.

#### Applications in Brain Tumor Surgery

Indocyanine green video angiography is a widely used method for intraoperative assessment of blood flow and vessel patency in tissue flap pedicles (81) and for assessment of intestinal perfusion near an anastomosis (82). ICG has been extensively studied for detection of sentinel lymph nodes in gastrointestinal oncology. Lymphatic drainage has been traced after subcutaneous ICG administration (83).

Although we do not discuss ICG angiography for vascular neurosurgery here, assessment of arterial and venous anatomy during some tumor resections may be necessary (84). Confirmation of the distal circulation with ICG angiography and test occlusion may be used when arterial sacrifice is required during tumor removal (85).

During a glial tumor resection using the operating microscope, ICG injection shows increased blood flow in the tumor tissue and pathology-induced alteration in the surrounding brain circulation (86). Hansen initially showed that ICG was able to highlight glioma tissue using an *in vivo* rat model (87), but therapeutically adequate doses did not produce sufficient fluorescence and required enhanced imaging technologies beyond the standard operating microscope (88, 89). Prior administration of bradykinin analog reportedly increased ICG extravasation and staining of tumor tissue in a glioma model (90) but not in the clinical setting. Simultaneous application of ICG during 5-ALA-guided glioma resection permitted detection of hypervascularized, angiogenic hotspots at the edge of resection potentially increasing the extent of resection (67). Although ICG produces an NIR signal with deeper penetration, it may not be specific for glial tumor tissue. ICG follows proteins leaked from the disrupted BBB and may diffuse into surrounding tissues. A unique liposomal formulated phospholipid-conjugated ICG has a particular brain-to-tumor biodistribution that may allow more accurate imaging guidance during surgery than ICG alone (91). Using a hand-held confocal endomicroscope, we observed that ICG selectively stained glioma cells *in vivo* (66).

Hemangioblastomas are highly vascularized tumors, and ICG fluorescence helps to identify hidden arterial feeders and vessels en passage (80, 92, 93). ICG has shown some usefulness in meningioma surgery. In cases of an occluded superior sagittal sinus, ICG was helpful in guiding the dural opening, tumor resection, and venous management, although multiple ICG injections were necessary (94). Additionally, ICG was used to highlight pituitary adenoma tissue through a microsurgical approach using the operative microscope (95, 96). ICG imaging using an endoscope was also recently reported to assist in visualization of tumors that infiltrated the sphenoid sinus (97) and to assess blood flow to the optic nerves and normal pituitary tissue during transsphenoidal surgery (98). ICG has also been used to guide transventricular endoscopic biopsies but not all areas of tumor dissemination were visible (99). ICG was further applied to visualize the facial nerve during temporal bone resection (100). After the facial canal was drilled to make it thin, ICG was injected and the fluorescence from the nerve blood supply guided further bony dissection to the internal acoustic meatus. Thus, the technique allowed visualization and preservation of the facial nerve (100).

One of the drawbacks of ICG visualization is that the image can only be displayed on a monitor, and technical refinements are needed to increase the comfort and ergonomics of ICG imaging instrumentation. Recent advances in this method include an overlay of fluorescence video angiography with a white-light field transmitted from the conventional operating microscope (101).

### 5-Aminolevulinic Acid

#### Characteristics

5-Aminolevulinic acid is a drug that is an intermediate metabolite of the heme synthesis pathway. 5-ALA is converted to protoporphyrin IX (PpIX), which is an endogenous fluorophore. PpIX peaks in 6 h after 5-ALA administration (102). Established correlation of gadolinium, a marker of BBB breakdown, with PpIX concentrations in glioma tissues, suggests (103) that BBB disruption is the leading cause of increased 5-ALA accumulation in malignant cells. However, increased 5-ALA-induced PpIX fluorescence was demonstrated within the areas with preserved BBB (104). PpIX has an excitation peak in the violet–blue light range (405 nm). Under blue light illumination, normal brain tissue reflects the light, whereas tumor tissue with accumulated PpIX emits a bright red fluorescence with two peaks, a large peak at 635 nm and a small peak at 710 nm.

Interest in 5-ALA application in neuro-oncology has been stimulated by promising PDT results with 5-ALA as the photosensitizer for the treatment of other types of cancers. PDT is recognized as a treatment modality mainly for tumors of hollow organs such as the stomach, colon, rectum (105), and most successfully, for skin malignancies (106). Its success is mainly related to a tumor location close to the surface that allows for sufficient depth of penetration by the irradiation.

#### Applications in Brain Tumor Surgery

For wide-field fluorescence, 5-ALA is usually administered 3 h before surgery so that the peak of PpIX production corresponds to the intraoperative tumor removal stage. Fluorescence observed in glioblastomas is often patchy and varies in intensity. Low-grade gliomas may not be visualized with wide-field techniques, although confocal endomicroscopy may detect 5-ALA in such tumors (107). In meningiomas, the observed fluorescence is usually high in intensity and is homogeneous (108). Tumor-specific fluorescence suffers from photobleaching. Natural fading occurs about 9 h after administration. 5-ALA-induced fluorescence decays to 36% of the peak within 25 min in light filtered to match the excitation wavelength of 405 nm; in contrast, with unfiltered wide-field illumination, 87 min was required to reach the same level of decay (25).

Most studies on glioma surgery with 5-ALA fluorescence for guidance have documented increases in tumor resection area (109, 110). The results of a phase III study indicated a 1.5-month increase in progression-free survival with 5-ALA fluorescence-guided surgery (111). In patients older than 55 years, regardless of tumor location, progression-free survival increased an additional 6 months. 5-ALA was also successfully used in brain tumor biopsy to obtain specimens of higher quality and to make a preliminary photodynamic diagnosis in a situation of primary central nervous system lymphoma (112).

Several approaches have advanced 5-ALA technology. One approach is to calculate the severity of the malignancy based on the fluorescence intensity. The emission spectrum must be analyzed accurately to calculate the ratio of peak emission intensity to the reflected excitation intensity (i.e., fluorescence intensity ratio). This ratio can then be used to predict the proliferative activity of the tumor (113). However, investigation of this characteristic was done in *ex vivo* tissue and requires technical improvement for intraoperative use. Other potential significant advancements for the use of 5-ALA involve the intraoperative use of high-magnification imaging optical technologies, such as confocal endomicroscopy, which may bring detection of fluorescence to the cellular level (107). The results of conventional histopathological methods correlated with confocal endomicroscopic imaging during 5-ALA-guided tumor resection (107). However, image quality was poor, and *in vivo* visualization of 5-ALA using blue laser confocal endomicroscopy in animal models could not confirm the findings (64, 107). Results from various clinical trials using intraoperative 5-ALA for brain tumor resection are ongoing (114).

5-Aminolevulinic acid, like all fluorophores, has drawbacks. Disruption of the BBB is necessary for fluorophore accumulation. In some low-grade gliomas, this may decrease or vary contrast accumulation. However, recent quantitative measurement studies suggest that diagnostic concentrations of PpIX do accumulate in low-grade tumors, but the concentration is below the detection threshold of current wide-field systems (115). Blood and overlying soft tissues can decrease visible fluorescence and hide the residual tumor. 5-ALA consumption and PpIX production may be highly variable (116) and depend on the several factors such as cell type (117), glucose concentration (118), pH (119), and other factors.

### Fluorescein

#### Characteristics

Fluorescein is an orange–red powder with the molecular formula C_20_H_12_O_5_ and a molecular weight of 332.31 Da. It is widely used in the scientific and medical industries as fluorescein isothiocyanate 1 (FITC), Alexa 488 fluorophore, and other variants. In medicine, the fluorescein sodium salt is used, but for brevity, we refer to it here as fluorescein. Fluorescein as a marker of BBB disruption demonstrated perilesional edema in a cortical cold lesion model in rats (120). Tumor boundaries observed using fluorescein fluorescence correlate well with preoperative gadolinium contrast-enhanced boundaries (68). However, fluorescein has no particular interaction with the tumor cells and may not show fluorescence in diffuse, low-density tumor cell infiltrates (68, 121, 122).

#### Application in Brain Tumor Surgery

Although the first clinical use of fluorescein for glioma surgery was in 1948 (20), fluorescein use in brain tumor surgery is not currently an FDA-approved use. Thus, it is restricted to clinical research studies. Its application is reported at several dosages. High doses of 15–20 mg/kg have been used for naked eye guidance without creating any permanent adverse effects (123). Yellow staining of the sclera, skin, and urine after high doses disappeared in approximately 24 h (124). Lower doses of 5–10 mg/kg for fluorescein-guided surgery using a special operative microscope module with excitation and observation filters were typically safe and effective in clinical trials (125), although one case of intraoperative anaphylaxis has been reported (126). The timing of fluorescein injection also varied across the studies. Some researchers have injected fluorescein intravenously after induction of anesthesia (127) at a dose of 3–4 mg/kg and waited for 10 min or 1 h, whereas others have injected it into a central venous line and waited for 20 min before resection (20, 124, 128–130). The half-life of fluorescein glucuronide, the main metabolite of fluorescein, is 264 min (131), and urinary clearance requires 24–32 h.

Fluorescein accumulates in glioma tissue homogenously and may be observed by the naked eye as bright to dark yellow staining of the tumor (123). Fluorescein-guided resection using operative microscopy without a special fluorescence detection module was reported by Shinoda et al. They achieved a gross total resection (GTR) in 84% of patients (129). Koc et al. produced GTR in 83% versus 55% of controls (132), and Chen et al. achieved an 80% GTR rate (123). The study by Koc et al. did not show a difference in survival (43.9 weeks in the patients given fluorescein and 41.8 weeks in the control patients) (132) while others did not assess survival.

A custom microscope for fluorescein-guided surgery was described in 1998 that increased fluorescent enhancement and contrast of intravenously injected fluorescein (8 mg/kg) during tumor removal (128), although there was diminished fluorescence in gadolinium unenhanced areas (65, 131, 133). The introduction of special filters to neurosurgical operative microscopes has stimulated interest in fluorescein-guided surgery, despite the dispute over fluorescein specificity for identifying tumor tissue.

Fluorescein has been used with success to guide removal of skull base tumors such as pituitary adenomas, craniopharyngiomas, meningiomas, and schwannomas (130). da Silva et al. reported enhancement of a meningioma dural tail by fluorescein (134). However, not all brain metastases and not all tumor areas were selectively highlighted by fluorescein, and some residual non-fluorescent tumor tissue was confirmed on postoperative enhanced MRI (135, 136).

Disruption of the BBB is an essential factor determining fluorescein extravasation, and several other factors may also confound fluorescein-guided glioma surgery. Variations in dose and timing of fluorescein administration may result in a variable degree of fluorescence in line with other factors such as fluorescein extravasation in surgically perturbed tissues, brain swelling, and unknown fluorescein distribution (127). Simultaneous administration of 5-ALA and fluorescein has shown that fluorescein was visible in normal brain and not detected in some areas highlighted by PpIX fluorescence (137). Thus, the benefit of fluorescein in guiding resection of malignancies is openly questioned and actively discussed. Some researchers warned that fluorescein application outside of clinical studies is premature and emphasized possible false positive and false negative staining during surgery (135). Nonetheless, confocal endomicroscopy with fluorescein in patients with brain tumors has revealed promising results on par with frozen section pathologic examination as a means of optically interrogating tissue (121).

### Other Fluorophores

Various new fluorophores and smart-targeted fluorescent probes are in different stages of preclinical development. Here, we review new fluorescent labels and activity-based and targeted bioengineered fluorescent probes.

Cresyl violet, acridine orange, and acriflavine are fluorescent dyes that were investigated for *ex vivo* use for rapid brain tumor tissue diagnosis using confocal endomicroscopy (64, 138). Methylene blue was used as a dye to color insulinomas and parathyroid glands to a blue hue. When diluted, methylene blue also acts as a 700-nm fluorophore and was studied for use in parathyroid (139) and breast tumor surgery (140). Methylene blue was administered at a dose of 1.0 mg/kg over 5 min and imaged with the Mini-Fluorescence-Assisted Resection and Exploration (FLARE)™ system. Methylene blue is excreted by the kidneys and therefore was investigated as an NIR fluorophore to visualize the ureters intraoperatively (141). NIR imaging of meningiomas and low- and high-grade gliomas topically stained with 0.05 mg/ml methylene blue provided good, but not specific, delineation of tumor cells (74).

Demeclocycline (excitation/emission peaks at 458/529 nm) is a tetracycline antibiotic with phototoxic effects. It has been used to demarcate tumor cells when used as an *ex vivo* stain on various human cancer tissues including gliomas (72, 142).

Novel cancer-selective alkylphosphocholine analog fluorophores CLR1501 (green with excitation/emission peaks 500/517 nm) and CLR1502 (NIR with excitation/emission peaks 760/778 nm) were reported to have higher tumor-to-normal brain fluorescence than 5-ALA (7.23 ± 1.63 and 9.28 ± 1.08 vs. 4.81 ± 0.92, respectively) in a mouse xenograft glioblastoma model (69). Another new fluorophore, a synthetic organic molecule CH1055 (970 Da), has a superior depth of penetration of almost 4 mm due to the higher emitted wavelength of about 1050 nm (70). High probe uptake by brain tumors in mice, the possibility of conjugation with anti-epidermal growth factor receptor (EGFR), and a high tumor-to-background ratio are reported. Combining the fluorophore with a radioactive probe is another promising surgical method for finding sentinel lymph nodes or residual tumor tissue that are deep and do not produce visual fluorescence (143), although it may not be useful for brain imaging because scintigraphy does not have the precision required for brain surgery.

#### Activity-Based Probes

Several new types of probes referred to as activity-based probes, “activatable” probes, fluorescence-quenched probes, or substrate-based probes were recently designed and investigated in preclinical studies (144–154) and recently reviewed in detail (155). Such probes contain a fluorophore that is “quenched” until the probe is activated (unquenched) by the given local environment. In caged-type fluorophores, some modified hydroxymethyl rhodamine green (Ac-HMRG; emission peak, 521 nm) probes are highly fluorescent when the quenching part of the probe is cleaved by its specific enzyme (57). One tumor-labeling strategy is to use quenching agents that are reconfigured by tumor-associated proteases, which are highly expressed in malignant tumor cells aiding invasion. For example, the matrix metalloproteinase-750 probe is activated by the broad range of matrix metalloproteinase family enzymes and facilitates accurate detection and complete removal of breast cancer tissue (58). One of the major drawbacks of untargeted probes, including activity-based probes, is their susceptibility to washout (active or passive removal from the site). One probe designed to be unquenched by cathepsin L and further covalently bound to protease reduced this limitation (152). Other limitations such as topical application of probes with inherent waiting time for binding, unknown biodistribution, and possibly uneven penetration await investigation. The practicality of fluorescence-guided surgery dictates that fluorescent tags should penetrate or bind to the cancer cells or remain in proximity to them for long enough to detect them.

One interesting approach is the design of activity-based probes such as caspase-sensitive nano-aggregation fluorescent probe (C-SNAF) that microaggregate after cleavage by caspase-3 and -7 by intramolecular cyclization (60). Another exciting approach is the complex activity-based probe. The construct is synthesized by combining a cell-penetrating peptide that may be activated and a nanoparticle labeled with gadolinium and Cy5 fluorophore. This complex probe is taken up by the tumor cells after matrix metalloproteinases 2 and 9 cleave the peptide and activate its cell-penetrating domain. In a murine model, gadolinium allowed *in vivo* visualization of the tumor using MRI and Cy5 allowed fluorescence detection (156). The advantages of such probes are the ability to carry several labels for various types of detection, high contrast to the background, and applicability to a wide range of tumors. However, this model was not tested for application in brain tumors.

#### Molecular Targeted Probes

Molecular targeted probes are also known as affinity-based probes. Targeting molecules with colored and fluorescent dyes has revolutionized microscopy. Application of this method of visual guidance for tumor resection is under investigation in cell cultures and animals by several research groups (149–154, 157, 158). Many known tumor targets such as EGFR, HER2, CD105, VEGFR, and folate receptors have been tested for fluorescence visualization of tumors. Additionally, targeting and highlighting of normal peripheral nerves have been investigated to prevent nerve injury during surgery (159).

Molecular targeted probes may be classified based on the fluorophore, targeted molecule, and other components. The majority of the targeting molecules fall into three categories:

1. Antibodies;
2. Recombinant antibody mimicking binders:
  - Affibodies: small (6.5-kDa) single domain engineered proteins that bind target proteins, imitating antibodies (160).
  - Nanobodies: a single variable domain of an antibody, which is capable of specific binding (161).
3. Aptamers: short single strands of nucleic acids, which are capable of specific binding (162, 163).

The rapid growth of targeted molecular probes has occurred because of the development of new fluorophores that may be conjugated to a variety of specific targeting molecules. Numerous possible combinations, including the possibility of adding a second (or more) label, significantly increase this potential. Many fluorophores have become commercially available and are being investigated in numerous preclinical and several clinical trials. Two clinical trials of IRD 800CW-labeled probes for visualization of breast cancer and familial adenomatous polyposis have been completed (164, 165), and other trials are recruiting patients (166–172) for the use of the Cy 5.5-labeled probe (173). However, none of these trials involve brain tumors. A promising fluorescent label, zwitterionic NIR fluorophore, ZW800-1, was recently described (50). ZW800-1 has great promise as it shows a higher tumor-to-background ratio than IRDye800-CW and Cy5.5 *in vitro* and *in vivo* (17.2 vs. 5.1 and 2.7, respectively) (50, 51).

The major drawbacks of targeted molecular probes are uneven passive distribution and non-specific binding. Dual-labeled probes were designed to address this limitation (174). This approach uses targeted probes that bind, and untargeted probes that do not bind, to the target. Differentiation in the fluorescence intensity allows a quantitative assessment of the binding potential of the probe (175, 176). Another drawback is that tumor regions with an undisrupted BBB can decrease the accumulation of the large probes. Sexton et al. addressed this problem by designing a small targeted fluorescent affibody peptide (about 7 kDa vs. 150 kDa for a full antibody) and demonstrated in a mouse xenograft GBM model almost two times increased fluorescence at the tumor edge compared to the full anti-EGFR antibody probe (42). Gong et al. showed that both anti-EGFR-specific affibody and the therapeutic antibody panitumumab labeled with IRDye 800CW could be used as imaging agents for both wild-type EGFR and EGFRvIII glioblastoma cells in cell culture studies (177).

In a 2014 report, Ghosh et al. described a novel targeted probe construct containing a single-walled carbon nanotube as a fluorescent tag (52). It consists of an M13 bacteriophage as a scaffold, a targeting protein, and the fluorescent nanotube. The single-walled carbon nanotubes have emission within the NIR range (950–1400 nm), resulting in less optical scattering and deeper tissue penetration. This setup is also less susceptible to photobleaching or quenching effects. The construct was stable and showed 10 times more selective fluorescent staining of ovarian tumor cells than the same construct without the targeting peptide. The nanotube fluorescence intensity ratio relative to the background (5.5 ± 1.2) was superior to the same construct labeled with other NIR AlexaFluor750 dye (3.1 ± 0.42) or FITC (0.96 ± 0.10). However, this study did not assess the possible penetration of the probe into the brain (52).

A targeted probe consisting of fluorescent gold nanoparticles conjugated with diatrizoic acid and AS1411 aptamer (53) has an absorption band of 300–400 nm and orange–red emission (maximum 620 nm), which can be observed by the naked eye. This probe showed specific binding to tumor cells due to the AS1411 aptamer, which targets nucleolin. The probe allowed X-ray visualization due to the high electron density of the gold nanoparticles. A novel lymphoma-specific fluorescent (Alex488) switchable TD05 aptamer in a human brain tumor xenograft model has been described (54). This probe rapidly and precisely identified human B cell lymphoma in biopsies. Such a system would be useful for rapidly discriminating non-operative CNS B-cell lymphoma from malignant glioma based on the biopsy.

Another agent, BLZ-100, a tumor ligand chlorotoxin conjugated to ICG, was shown to have high affinity to human gliomas in mice (55). Chlorotoxin was extensively studied in preclinical *in vivo* studies as a conjugate with Cy 5.5 (178) and IRDye 800CW (179). Chlorotoxin is a new drug that binds to chloride channel–MMP-2 membrane complexes. It reduced the invasiveness of glioma cells (180), inhibited glioma cell growth and metastasis, and accelerated tumor apoptosis (181). The advantage of the probe is its small size and ability to penetrate the BBB.

Another 5-carboxyfluorescein-labeled fluorescent probe consisting of tLyP-1 small peptide targeting neuropilin receptors was recently described. Neuropilin receptors are co-receptors for vascular endothelial growth factor and play a role in tumor-mediated angiogenesis. They are overexpressed in most gliomas. The probe has selective uptake and may have advantages over the CTX-Cy5.5 probe due to its small size. However, the fluorescein labeling was less than ideal and could be exchanged for a more intense fluorophore for use in intraoperative imaging (56).

IRDye800CW-labeled anti-EGFR nanobody 7D12 was compared to the full antibody cetuximab and showed better penetration and distribution of the nanobody probe *in vivo* in a preclinical study (40). Another study of the same nanobody for orthotopic tongue tumors showed significantly higher tumor to background fluorescence (2.00 ± 0.34 in the FLARE imaging system) than in the group with the same non-targeted fluorescent probe (41).

Polyacrylamide nanoparticles have been coated with the F3 protein that binds to nucleolin and loaded with methylene blue, Coomassie blue, or ICG. F3-coated constructs increased the color change in glioma cells *in vitro* (61). Magnetic NH_2_-cross-linked iron oxide nanoparticles labeled with Cy5.5 (32 nm in diameter) produced clear tumor border demarcation and co-localization on MRI imaging in a rat gliosarcoma model (62). As a non-targeted construct, it produced demarcation mainly due to BBB disruption and was designed as a magneto-optical probe. Additionally, iron oxide particles are eliminated by reticuloendothelial cell endocytosis (182), suggesting that their elimination is more predictable than that of other nanoprobes. However, this magnetic nanoparticle design is less attractive than that of targeted probes, which aim to increase the accuracy of tumor cell visualization *in vivo*.

Summarizing fluorophore use in neurosurgery, 5-ALA-guided brain tumor surgery may improve the gross tumor resection rate and is approved in Europe but is available only in clinical trials in the US. Fluorescein-guided resection has emerged as an alternative due to its safety profile, although fluorescein is not tumor cell-specific. ICG shows promise for vascular tumors, such as hemangioblastomas, but may also have the potential to define malignant gliomas. Many new targeted and activatable fluorescent probes are awaiting full assessment to be used in clinical studies. Although molecular targeting probes are attractive and technologically advanced, their benefit and cost compared to already existing 5-ALA and fluorescein for fluorescence-guided resection are yet to be proven. Assessing the advantages of the many probes being designed is a difficult and time-consuming task considering the emerging improved, quantitative fluorescent detection methods. Combining the probes with molecules for secondary goals such as chemotherapy, photosensitization, and others may be advantageous.

## Instrumentation for Fluorescence-Guided Resection

Several different technologies are applied in fluorescence-guided resection of brain tumors. These technologies are classified into several categories (183–187):

1. Wide-field fluorescence imaging:
  - Commercial operative microscopes with built-in fluorescence channels;
  - Custom modified surgical microscopes;
  - Surgical endoscopes equipped with fluorescence modules;
  - Non-microscope fluorescent excitation systems with emission detecting devices.
2. Quantitative fluorescence systems:
  - Spectroscopic tools for imaging one region at a time;
  - Laboratory grade stand-alone systems;
  - Combination systems that integrate fluorescence with spatial imaging.
3. Intraoperative high-resolution endomicroscopy.

### Wide-Field Fluorescence Imaging

Wide-field fluorescence imaging refers to non-microscopic, endoscopic, or microsurgery in which full fields of view are seen continuously through the eyepieces or on the screen during image acquisition at a rapid frame rate with a digital detector array (CMOS or CCD cameras) (184). Several instrument solutions exist for wild-field fluorescence imaging. By definition, such systems have a magnification of 5× to 40× and resolution of less than cellular level. Fluorescence-guided surgery is undergoing revitalization as advancements in optics are allowing improved visual perception of fluorescence. Numerous neurosurgical studies highlight the benefits of wide-field fluorescence-guided glial tumor resection, mainly to increase the GTR rate and progression-free survival (2, 111, 188). Some studies even showed increased overall survival (189).

#### Instruments

**Figure 2:**
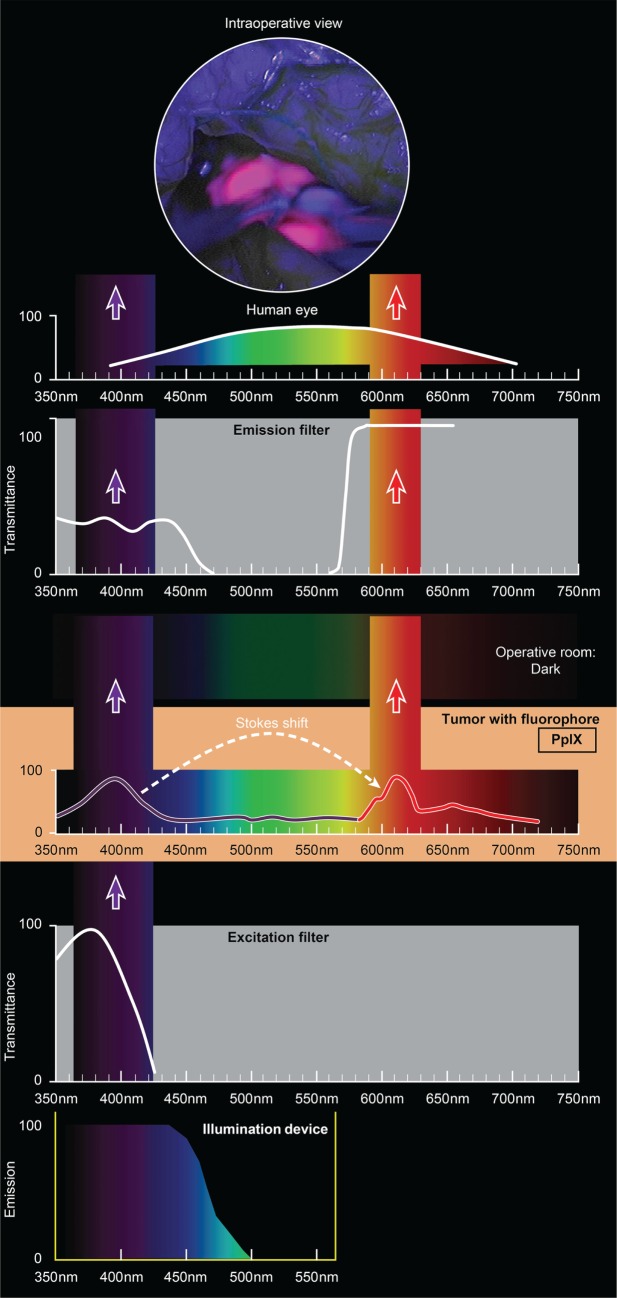
**Schematic view of the concept of PpIX-guided tumor visualization using a wide-field operative microscope with appropriate filters**. Wavelength scales are in the same position in the figure. The illumination device emits light in the wavelength band less than 470 nm. The excitation filter then transmits light with the peak of about 405 nm. PpIX, which is accumulated in the tumor cells, absorbs photons in the spectrum band around 405 nm and then emits photons of lower energy at a wavelength of about 630 nm. The blue light from the illumination device and the emitted red fluorescence band are observed through the operative microscope optics equipped with an emission (observation) filter. This filter has a cut-off transmittance at about 450 nm and cut-on transmittance at about 570 nm. The two bands of light observed fall into the visible spectrum (with the naked eye) and are perceived as a violet–blue background and “pink-to-red” fluorescence. The light in between those two bands is blocked; therefore green, yellow, and orange colors are not visible. PpIX, protoporphyrin IX. Used with permission from Barrow Neurological Institute, Phoenix, AZ, USA.

**Figure 3:**
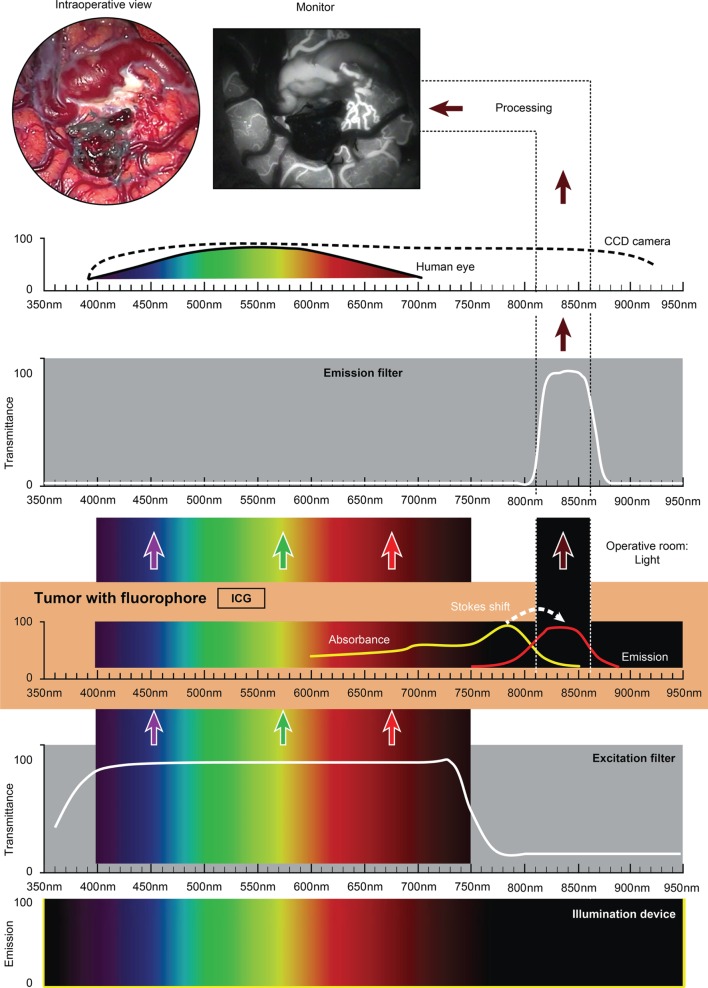
**Schematic view of the concept of ICG fluorescence visualization using a wide-field surgical microscope with appropriate filters**. Wavelength scales are in the same position in the figure. The illumination device (xenon lamp) emits light in a wide range of wavelengths. The excitation filter cuts off the light longer than about 750 nm. ICG present in the tissue (vessels) absorbs photons in the available spectrum band below 750 nm and then emits photons in a NIR spectrum around 820 nm, invisible to the naked eye. The emission filter then transmits this NIR light to the CCD camera and blocks the light with other wavelengths. The CCD camera records the images during the desired period. After image processing, the resultant surgical picture is displayed on the monitor of the neurosurgical microscope in the grayscale as a short movie fragment. ICG, indocyanine green. Used with permission from Barrow Neurological Institute, Phoenix, AZ, USA.

**Figure 4:**
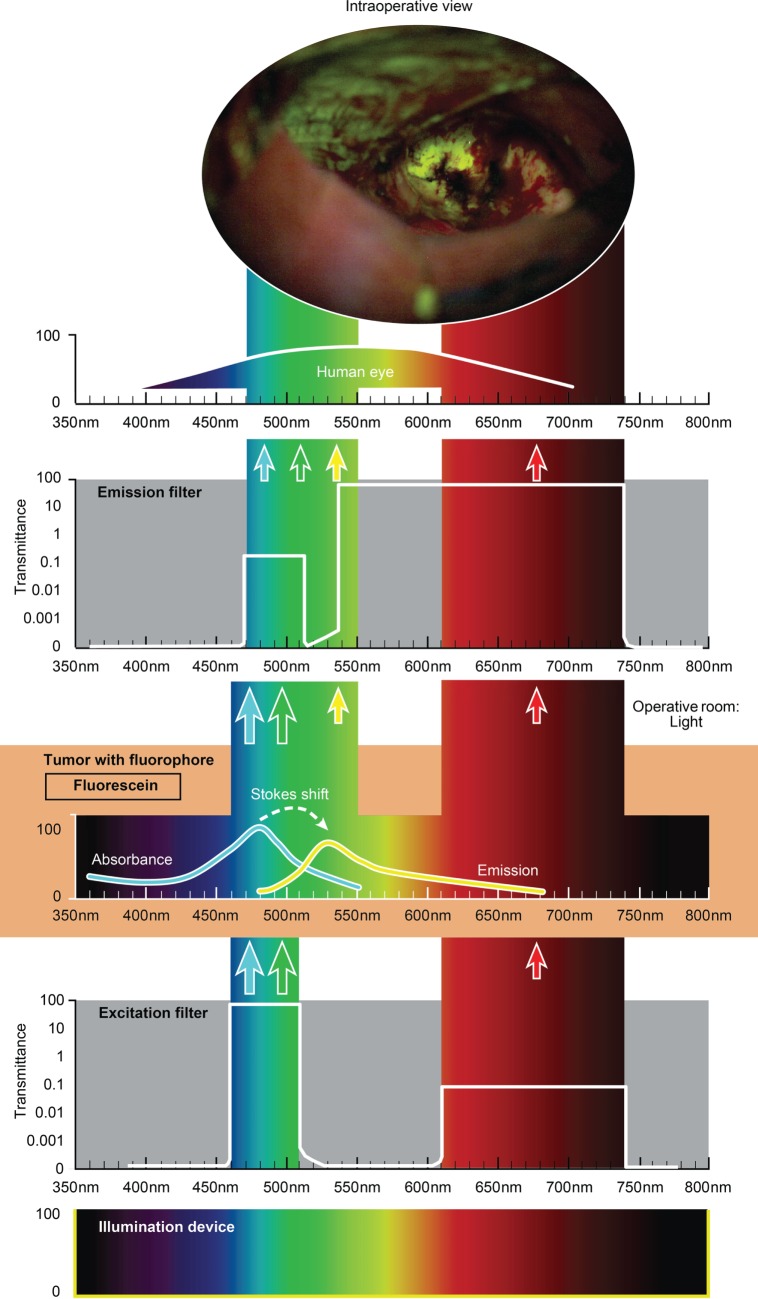
**Schematic view of the concept of fluorescein-guided tumor visualization using a wide-field operative microscope with appropriate filters (https://www.google.ch/patents/US8730601)**. Wavelength scales are in the same position in the figure. The illumination device (xenon lamp) emits light in a broad range of wavelengths. The excitation filter then transmits the light as narrow bands at about 450–520 nm and about 600–750 nm. The first (blue–green) transmittance band is significantly more intense (see log scale on the side of the filters in the figure) than the second (red) band of light. Fluorescein, which is accumulated in the tumor tissue, absorbs photons in the spectrum band around 485 nm (high-intensity band) and then emits photons with a wavelength around 514 nm (yellow) with a lower energy (new low-intensity yellow band). Blue–green and red bands of light from the illumination device, as well as the new yellow (around 514 nm) fluorescence band, are observed through the operative microscope optics equipped with an emission (observation) filter. This emission filter has a transmittance in two bands: first in the range of 475–515 nm with significantly lower transmittance (see log scale in the figure) and the second in the range of 530–700 nm with the maximum transmittance. The three bands of light, the blue–green emission band, red band, and emitted yellow band, all fall into the naked-eye-visible spectrum for observation. The transmittance of all filters together results in the uniform intensity of all bands, with a higher possible intensity of emitted yellow light. A portion of the spectrum between the bands could be blocked by the filters, but the remaining three primary color bands allow the surgeon to see the intraoperative picture with almost the full spectrum of colors. Used with permission from Barrow Neurological Institute, Phoenix, AZ, USA.

The use of custom operative microscopes with modules to measure fluorescein (128) and PpIX (190) fluorescence was reported in 1998. There are three fluorescence detection modules, for use on commercially available operative microscopes for 5-ALA- (Figure 2), ICG- (Figure 3), and fluorescein-guided (Figure 4) tumor resection in the brain. The modules consist of three components: (1) a set of optical filters for selective wavelength separation, (2) a broad-spectrum illumination device, and (3) optional CCD cameras for detection of visible and invisible (NIR) light (Figure 3). New optical filters for selection of bandpass width and blocking intensity are now available due to progress in optical engineering. A combination of these new filters permits the best possible fluorescence intensity and contrast to the background ratio. One example is the new Yellow 560 Module for the Zeiss operative microscope that reintroduced fluorescein in brain tumor surgery. A set of filters facilitates excitation of the fluorescein with the maximum intensity while preserving illumination of the background with another visible spectral band of lower intensity (Figure 4). The resulting bright yellow fluorescence of tumors is observed and contrasted with the natural colored background (191).

Fluorophores that emit in the NIR spectrum require CCD cameras or other detection technologies. An ICG (NIR) module does not require an operative microscope *per se* because the resultant fluorescence is not perceived by the eye and is observed on an ancillary screen. The fact that NIR probes are in abundance and are either commercially available or awaiting approval by the FDA (ICG, Cy 5.5, IRDye800-CW, and BLZ-100) has stimulated the field of computer engineering to develop wide-field systems that overlay an NIR signal on the surgical field of view. Such an overlay is desirable in real time and with measurable specificity. Non-microscopic, mobile, wide-field video imaging systems for open, laparoscopic, thoracoscopic, and robotic surgery are in development and clinical trials (192, 193).

Work in intraoperative NIR imaging technologies in neurosurgery shows potential for advantageous applications. A novel proof-of-concept NIR imaging system consists of a narrow-band laser at 785 nm, a notch filter, and a standard 2-CCD camera for wide-field visualization. This system has been tested with an ICG-conjugated targeted BLZ-100 probe in a murine brain tumor model (55).

A new endoscopic technology, scanning fiber endoscopy (194), also holds great promise due to its ultrathin probe and increased resolution. A color image is acquired by combining red, blue, and green laser lights through a spiral actuated optical fiber. Laser induced fluorescent imaging of 5-ALA-induced PpIX fluorescence on tumor cell phantoms (195) and a murine tumor model (work in progress) allowed detection of the fluorescence with greater sensitivity than through the operative microscope. We found this optical imaging technology very convenient for potential intraoperative use due to its small size with a field of view of 2–30 mm and high spatial resolution of up to 15 μm.

A concept for a low-cost fluorescein detection system for glioma surgery (196) consists of a xenon light source, fiber optic light cable, a set of glass interference filters (neutral, 490 nm, 465 nm), and yellow photographic filters for oculars or UV yellow glasses. In clinical trials, the system showed great potential due to its low cost, especially beneficial for low-income countries, although limitations of the custom hardware and fluorescein usefulness in glioma surgery itself still require confirmation (127).

Limitations of wide-field visualization technologies in fluorescein-guided surgery are similar to those of 5-ALA studies. Wide-field, fluorescence-guided surgery limitations include (184) ambiguity at the margins where fluorescence intensity decays and difficulty of visualization on the sides of a resection cavity and shaded areas in the surgical wound. Further limitations include fluorescence absorbance by blood and tissue layers (197), insufficient fluorescence intensity in >95% of low-grade gliomas (107, 198, 199), and lack of quantitative assessment of fluorescence intensity. In PpIX fluorescence visualization, PpIX is quantitatively related at the microscopic level to increasing malignancy in both low- and high-grade gliomas (117). Such works emphasize the limitations of fluorescence detection by the current wide-field technologies at low concentrations of the fluorophore or when few cells are labeled. New approaches that increase the sensitivity of visualization systems include the use of a quantitative spectrophotometer, an additional camera for quantitative image processing (200), and new endoscopic and confocal endomicroscopy probes.

### Intraoperative Quantification of Fluorescence

Absorption, scatter, anisotropy, and autofluorescence of the tumor and background tissue play important roles in the detection of the fluorophore signal, especially at low signal levels. Thus corrections for the optical properties of the tissues provide qualitative information about the fluorescence intensity in the area of interest (193). For this reason, researchers have studied the utility of spectrophotometric quantification of PpIX emission spectra. Visual light spectroscopy for the calculation of the background fluorescence intensity ratio has been investigated in postoperative astrocytoma samples (113). The fluorescence intensity correlated with the MIB-1 proliferation index, a prognostic indicator for tumor progression.

A spectrally resolved quantitative fluorescence imaging system with submillimeter spatial resolution (214–125 μm) has been integrated with a conventional operative microscope (200). This system provides a colored digital overlay of the quantitative fluorescence intensity map over the surgical field of view. A pilot study of human glioblastoma surgery showed that the system showed a signal from histologically confirmed residual tumor tissue when the standard wide-field BLUE400 filter image was negative (200). Quantitative PpIX detection elevated the diagnostic sensitivity of low-grade gliomas (67% in 12 cases) to the level of qualitative wide-field detection of high-grade gliomas (115). Improved sensitivity with PpIX fluorescence was confirmed using an *ex vivo* animal tumor model with a similar system that included a CCD camera attached to the operative microscope (201). Quantification using the intraoperative contact optical probe demonstrated increased accuracy in detecting neoplastic meningioma tissue with a 90% diagnostic accuracy for differentiating tumor from the normal dura in 10 grade 1 meningiomas (108).

### Intraoperative Confocal Endomicroscopy

**Figure 5:**
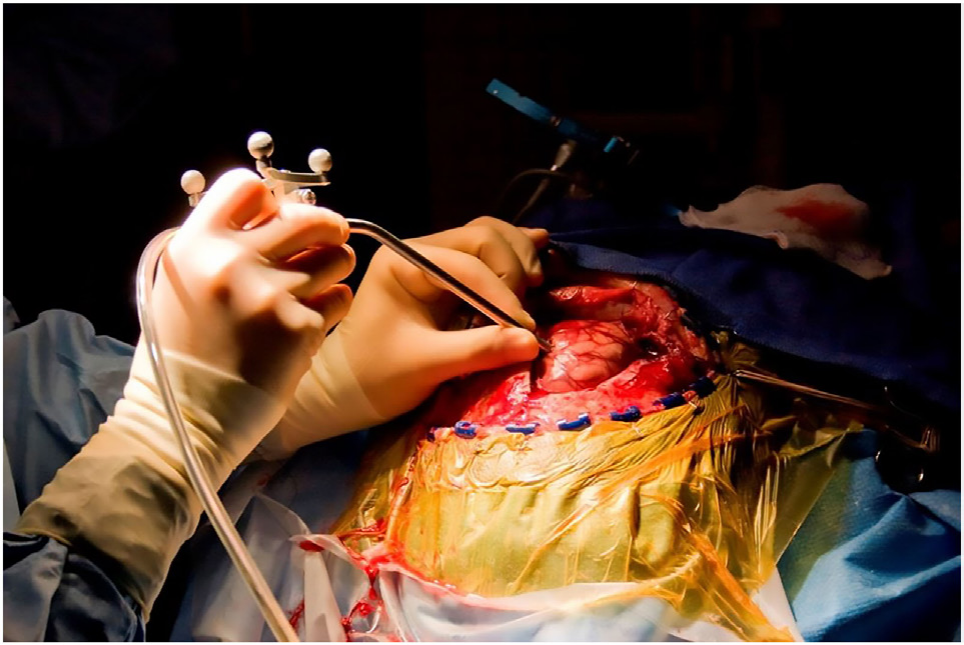
**Intraoperative use of a hand-held confocal endomicroscopy probe co-registered with a StealthStation neuronavigation system during brain tumor surgery**. Used with permission from Barrow Neurological Institute, Phoenix, AZ, USA.

**Figure 6:**
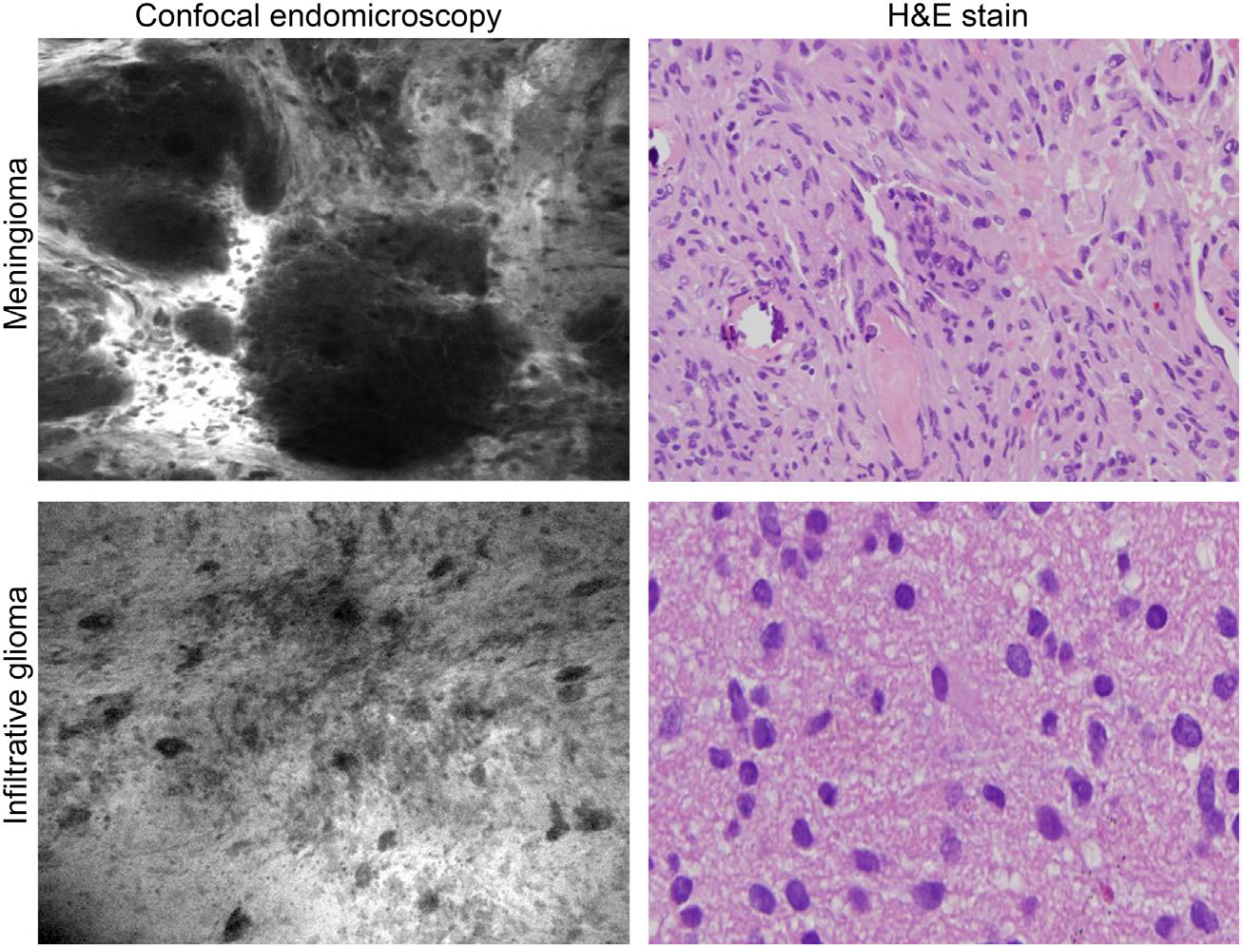
**Intraoperative images of meningioma and glioma after intravenous fluorescein sodium injection taken with the confocal endomicroscopy probe and shown with corresponding histopathological pictures**. Used with permission from Barrow Neurological Institute, Phoenix, AZ, USA.

The rationale for high-resolution intraoperative imaging is the inherent limitations of wide-field fluorescence microscopy and the desire for precise tissue visualization at the cellular level. Fiber optic confocal microscopy was invented in 1988 (202), commercialized in 1994, and the first results of use in neurosurgery were published in 2010 (122). Such systems consist of a miniature handheld probe and movable workstation with an LCD screen (Figure 5). Excitation and emission light is transmitted through a single optic fiber. The system provides non-invasive real-time imaging through optical sectioning at a known depth. Most importantly, it appears to provide real-time images for histopathological analysis without the laborious process of tissue preparation, although this development is still being validated (Figure 6) (203). Two commercially available systems include the Optiscan FIVE 1^1^ and Cellvizio.^2^ Both systems have been reviewed for neurosurgical applications (186). The Optiscan has a 475 μm × 475 μm field of view with a focal plane to a depth of 250 μm, and the Cellvizio has nine objectives covering fields of view ranging from 300 to 600 μm and 15 to 70 μm optical sectioning depth. The Cellvizio and Optiscan currently use a 488-nm excitation light, and the Cellvizio also has a 660-nm single-band excitation light. The first feasibility study of intraoperative confocal endomicroscopy was reported for a variety of brain tumor pathologies in 33 patients with intravenous fluorescein injection for tumor visualization (204). Intraoperative imaging permitted the neuropathologist to make a diagnosis, but this diagnosis was not compared with standard histological staining for accuracy (205). Another study using confocal endomicroscopy enabled the correct diagnoses based on intraoperative images (fluorescein) in 26/28 of cases (206). A clinical series of 74 patients who underwent intraoperative confocal endomicroscopy showed diagnostic specificity and sensitivity for gliomas of 94 and 91%, respectively, compared to the interpretation of frozen section and permanent histologic diagnoses (121). Ongoing studies of this technology aim to improve these indices.

Confocal endomicroscopy may also be employed as a rapid diagnostic tool for biopsy specimens in *ex vivo* tissue analysis within the operating room. The utility of fluorescein, 5-ALA, acridine orange (stains DNA/RNA/lysosome), acriflavine (topical application, stains membrane/DNA), cresyl violet (topical application, stains ER/cytoplasm), and sulforhodamine 101 (topical application, stains glial cell cytoplasm) for visualization of tumor cells was demonstrated with the Optiscan 5.1 system (64, 122).

Although 5-ALA visualization was not optimal due to the limitation of the probe excitation profile, the other fluorescent stains clearly showed the histological features of the tumor cells and margins in a murine brain tumor model. Normal morphology in various brain regions was also clearly discernible in a large animal model (pig) using confocal endomicroscopy with topical acridine orange. Selective detection of ICG in a murine glioblastoma model was also shown using a clinical-grade, NIR confocal endomicroscopic system (64).

The initial experience with the Cellvizio confocal endomicroscope for immediate *ex vivo* imaging of human intracranial tumors after fluorescein-guided resection combined with topical acriflavine staining shows practical potential (138). Although rapid histopathological diagnoses were possible for a wide variety of brain neoplasms, this application is pending comparison with standard histological staining in validation assessments. Clinical trials of the Cellvizio system for brain tumors are underway in Europe to assess the neuropathological diagnostic agreement and completeness of tumor removal (207–209). Furthermore, the confocal endomicroscope was successfully used to visualize targeted probes consisting of two tyrosinase-related protein antibodies labeled with Alexa Fluor 488 fluorescent dye (210). In a murine brain tumor model, these probes correctly identified tumor cells with high specificity, confirming in principle that the targeted probes could be used along with the confocal endomicroscope to increase the extent of resection in a variety of brain tumors (211).

The inherent limitations of the intraoperative confocal endomicroscope are a narrow field of view, the image appearing on a separate display, and the necessity of non-standard image analysis and interpretation, along with limited resolution, laser excitation spectrum, and corresponding detection power. Several computer image processing methods have been proposed to improve the diagnostic value of these small-field-of-view systems. For example, an image stitching technique has been applied to create panoramic wide-field images (212–214). Multiple histogram operations provide image contrast enhancement (215). Image quality and its diagnostic value, as well as the surgeon’s knowledge of histopathology, are important factors in the practical application of intraoperative confocal endomicroscopy because the resultant images differ from the stained histopathological slides and require additional training for interpretation. Additionally, the probe should be in a stable position during image acquisition, although the surgeon may acquire good images in the free-hand mode with practice.

Approval of targeted fluorescent probes for clinical use will likely stimulate the refinement of confocal endomicroscopy and its broad clinical use in neurosurgery and tumor pathology. These two technologies are complimentary and allow tailored, tumor-specific resections for personalized patient treatment and, certainly, precision tumor surgery. The ability to interrogate the tumor border optically is of significant advantage in the acquisition of selective biopsies of higher diagnostic yield. Such a situation could improve the neurosurgery–neuropathology workflow for increased efficiency.

## Future Directions

Several studies in optics, bioengineering, biotechnology, experimental oncology, and biochemistry have advanced the field of fluorescence-guided surgery in the preclinical arena (216). Because many fluorophores emit light in the NIR band, outside of the visible spectrum, improvements in overlay imaging technology are expected. Pharmacological and toxicological restrictions stimulate the application of “microdoses” of a fluorophore, which, in turn, may allow for approval for clinical use. Moreover, the fluorophores in use still require more sensitive detectors. The need for these features drives the focus of future system developments in fluorescence-guided surgical imaging and overlay techniques (216).

Pulsed-light imaging is a technology that exploits pulsed excitation light and time-gated detection. It allows fluorescence imaging under normal operating room light conditions with high detection sensitivity (217). This technology is more sensitive to lower concentrations of PpIX than surgical microscopy (217).

A novel type of fluorophore, quantum dots, appears to be a relevant nanotechnology for fluorescence. The quantum dot is a 5- to 20-nm nanocrystal made from a semiconductor material that acts like a traditional fluorophore but works by a different mechanism. The emitted wavelength of the quantum dot depends on the size of the crystal. Fluorescent probes with the desired emission band may be designed. The main advantages of the quantum dot are much longer excitation life leading to photostability. The color of the emitted light may be tuned to the size of the probe. However, the safety of quantum dots is significant because larger quantum dots may not be well cleared, and the long-term effects of accumulation are unknown (218, 219). Quantum dots conjugated with transferrin have been used as a fluorescent probe to target transferrin receptors in glioblastoma cells (220).

Another important parameter of future fluorophore probes is the size of the molecule, in which small targeted molecules, even with lower affinity, show better delineation of tumor boundaries most likely due to crossing the BBB more easily (42). In another approach, the BBB is reversely disrupted to allow more intense binding to the tumor tissue. Several methods to bypass the BBB were developed (221, 222) to enhance targeted fluorescent probe binding to brain tumor tissue and were tested in animals (63).

Some other emerging technologies may help in differentiating normal tissue from brain tumor tissue. For example, optical coherence tomography does not require any targeting agent. The technology utilizes differences in the optical signatures of the tissues to differentiate brain tumor from normal tissue, as shown in an animal study (223).

Intraoperative fluorescence imaging is capable of maximizing tumor tissue resection, providing rapid histopathological diagnoses based on innovative fluorophore probes and tools for intraoperative visualization. What is clear is that we sit on the threshold of technology that will enable neurosurgeons to see tumor cells in groups or individually in real time, which will allow tailoring or personalization of neurosurgery in terms of tumor resection. The term “theranostics” was coined to define ongoing efforts to develop precise, specific, individualized diagnostics and therapeutics for various diseases. For neurosurgery, we are adapting true precision modalities or biomarker techniques into diagnosis, including the imaging techniques described here, and this facilitates precise approaches to surgery. Cell-specific visualization will make possible the optimal surgical treatment of invading tumors such as gliomas that are composed of heterogeneous tissue with various genetic and metabolic characteristics. Therefore, the previously impossible may become routinely possible. If invading tumor cells are discovered in eloquent cortex, which is not normally resected, the neurosurgeon might be able to proceed on a cell-by-cell basis, targeting only tumor cells. Improved imaging technologies will bring about novel techniques to target or remove tissue or even individual cells. The advantages of such techniques are better surgical outcomes as nearly “cell-by-cell” or precision surgery becomes possible. Such surgical advancements will undoubtedly come with additional responsibilities, decisions, and challenges to be faced by both the neurosurgeon and patient.

## Author Contributions

All authors made substantial contributions to the conception or design of the work.

## Conflict of Interest Statement

This research is supported in part by Zeiss, but they did not take part in the design of the experiments, examination of data, or writing of the manuscript. The authors declare that the research was conducted in the absence of any commercial or financial relationships that could be construed as a potential conflict of interest.

## Footnotes

^1^www.optiscan.com

^2^www.maunakeatech.com

## Abbreviations

5-ALA, 5-aminolevulenic acid; BBB, blood–brain barrier; EGFR, epidermal growth factor receptor; FITC, fluorescein isothiocyanate; GTR, gross total resection; ICG, indocyanine green; NIR, near-infrared; PDT, photodynamic therapy; PpIX, protoporphyrin IX; ROS, reactive oxygen species.
